# Mapping health assessment questionnaire disability index onto EQ-5D-5L in China

**DOI:** 10.3389/fpubh.2023.1123552

**Published:** 2023-04-18

**Authors:** Chuchuan Wan, Qiqi Wang, Zhaoqi Xu, Yuankai Huang, Xiaoyu Xi

**Affiliations:** The Research Center of National Drug Policy & Ecosystem, China Pharmaceutical University, Nanjing, China

**Keywords:** HAQ-DI, EQ-5D-5L, mapping, Rheumatoid Arthritis, health utility, China

## Abstract

**Objective:**

This research aimed to develop the more accurate mapping algorithms from health assessment questionnaire disability index (HAQ-DI) onto EQ-5D-5L based on Chinese Rheumatoid Arthritis patients.

**Methods:**

The cross-sectional data of Chinese RA patients from 8 tertiary hospitals across four provincial capitals was used for constructing the mapping algorithms. Direct mapping using Ordinary least squares regression (OLS), the general linear regression model (GLM), MM-estimator model (MM), Tobit regression model (Tobit), Beta regression model (Beta) and the adjusted limited dependent variable mixture model (ALDVMM) and response mapping using Multivariate Ordered Probit regression model (MV-Probit) were carried out. HAQ-DI score, age, gender, BMI, DAS28-ESR and PtAAP were included as the explanatory variables. The bootstrap was used for validation of mapping algorithms. The average ranking of mean absolute error (MAE), root mean square error (RMSE), adjusted *R*^2^ (adj*R*^2^) and concordance correlation coefficient (CCC) were used to assess the predictive ability of the mapping algorithms.

**Results:**

According to the average ranking of MAE, RMSE, adj*R*^2^, and CCC, the mapping algorithm based on Beta performed the best. The mapping algorithm would perform better as the number of variables increasing.

**Conclusion:**

The mapping algorithms provided in this research can help researchers to obtain the health utility values more accurately. Researchers can choose the mapping algorithms under different combinations of variables based on the actual data.

## Introduction

Rheumatoid Arthritis (RA) is a chronic autoimmune disorder with symmetrical, recurrent, incurable and highly disabling (1, 2). RA has negative impact on patients physically, psychologically, and socially, such as leading to reduced daily activities, depression, changes in career plans, and reduced financial income, etc., which seriously affects the quality of life for patients (3). RA also imposes a huge financial burden on patients and society. Globally, RA imposes the greatest burden of all rheumatic diseases (4). In mainland China, the annual economic burden of RA patients is as high as 72 million Chinese Yuan (CNY). Considering the influence of per capita disability adjusted life years (DALYs), the annual economic burden is as high as 902 million CNY and annual economic burden per capita is 15,718 CNY (5).

Given the severe burden of RA and finite health resources, it is necessary to assess the value of interventions for RA. Cost-utility analysis (CUA) is the most widely used economic evaluation method (6), in which quality adjusted life years (QALYs) is adopted as the main health outcome (7). As an essential metric for calculating QALY, health utility value (HUV) is often obtained through a preference-based measure (PBM), like EuroQol Five-dimensions Questionnaire (EQ-5D), Short Form Six-dimension (SF-6D), Health Utilities Index (HUI) (8–10). As the most commonly used PBM, EQ-5D includes EQ-5D-3L, EQ-5D-5L, and EQ-5D-Y, with EQ-5D-5L being more widely used in China. In clinical, the health assessment questionnaire disability index (HAQ-DI) is widely used to assess the quality of life and function of RA patients (11). However, researchers can't get HUV of patient from HAQ-DI which is a non-preference-based measure.

Up to now, a large number of researches have demonstrated that the value of HAQ-DI can be converted into HUV through mapping (12–21). In Most researches, EQ-5D-3L was chosen as the target scale and the HAQ-DI total score as a single independent variable. Part of researches also would include sociodemographic or clinical characteristics as covariates. Interestingly, some of the CUA conducted on Chinese RA patients chose to use the mapping algorithm developed on Spanish populations (19, 22–24), although the mapping algorithms based on Chinese populations were available (13). Now, two researches have developed the mapping algorithms from HAQ-DI to EQ-5D-5L based on Chinese population. But they both suffered from single sample source, few models and variables selected.

Hence, we aimed to construct a mapping algorithm from HAQ-DI to EQ-5D-5L based on richer data sources, model selection and variable including. And the algorithm can be used to address the lack of HUV in health technology assessment for Chinese RA patients.

## Materials and methods

We adopted the cross-sectional data of RA patients, in which the EQ-5D-5L and HAQ-DI were used to measure and value HRQOL. The data were collected by trained investigators based on quota sampling during June to July 2020, which from 8 tertiary hospitals across four provincial capitals Nanjing, Hangzhou, Chengdu, and Shijiazhuang (two of each). This study complied with the principles of the Declaration of Helsinki and approved by the Clinical Trial Ethics Committee of Huashan Hospital Affiliated to Fudan University (Reference Number 2019-252).

### Sample

Given the available research resources and rules of thumb, 25 patients were recruited for each center, a total of 200 patients. The inclusion criteria were: (1) Informed and voluntary; (2) 18 to 70 years; (3) Diagnosed with RA according to the 2010 American College of Rheumatology (ACR)/European League Against Rheumatism (EULAR) classification diagnostic criteria (score ≥6) (25). The exclusion criteria were: (1) non-Chinese; (2) Gravidae and the patients who are unconscious; (3) Suffering from other serious diseases that seriously affect the quality of life, like tumors, myocardial infarct.

### Data collection

Data collection for each center was done by 2 interviewers who were systematically trained and knowledgeable about the content and methodology of research. The interviewers introduced the research to patients and doctors in the corresponding departments with the permission of the hospital administrator. For patients and their attending doctors willing to participate in the research, the interviewers would provide written informed consent to them. Then, the patients and doctors would be provided with an electronic device containing the research questionnaire and asked to complete the respective questionnaires independently in a quiet room without any guidance from the interviewers. The questionnaires completed were reviewed by the interviewers and uploaded to the auditors if no obvious errors or blanks. And the data would be digitalized and reviewed by 2 auditors.

### Questionaries

Questionnaires were designed for doctors and patients separately. The literature (26, 27) and experts' opinions were drawn upon. According to the results of a pilot survey conducted in 2 tertiary hospitals in Nanjing, we revised and formed the final questionnaires. The rationality, readability and comprehensibility for questionnaires had been affirmed by the experts and supported by the pilot survey.

The questionnaire had two parts for patients. Part 1 collected socio-demographic information, including age, gender, height, weight, region, education level. Part 2 collected some health status indicators which reported by patients through EQ-5D-5L, HAQ-DI, the patient's assessment of arthritis pain visual analog scale (PtAAP-VAS) and the patient's global assessment of disease activity visual analog scale (PtGADA-VAS). PtAAP-VAS and PtGADA-VAS were used to assesses arthritis pain and disease activity, of which 0 means no symptoms and 100 means severe symptoms.

The questionnaire also had two parts for doctors. Part 1 collected clinical information for patients, including high-sensitivity C-reactive protein (CRP) (unit: mg/L), erythrocyte sedimentation rate (ESR) (unit: mm/h), swollen joints count (SJC) and tender joints count (TJC). Part 2 collected the physician's assessment of the patients' disease activity through the Physician's global assessment of disease activity visual analog scale (PhGADA-VAS), of which 0 means no symptoms and 100 means severe symptoms. In addition, we calculated the 28 joint counts (DAS28) scores (28), including DAS28-CRP score and DAS28-ESR score, based on CRP, ESR SJC and TJC. Disease activity is divided into four states, including remission (DAS28 scores <2.6), low activity group (2.6 <DAS28 scores <3.2), moderate activity group (3.2 ≤ DAS28 scores <5.1), and high activity group (DAS28 scores ≥5.1) (28).

### EQ-5D-5L

The EQ-5D-5L is more sensitive compared with EQ-5D-3L and has been widely used to measure HUV. The EQ-5D-5L essentially consists of 2 parts: the EQ-5D descriptive system and the EQ visual analog scale (EQ VAS) (29). The descriptive system contains five dimensions, including three functional dimensions (mobility, self-care, and usual activities) and two somatic symptom dimensions (pain/discomfort, and anxiety/depression), each of which is divided into five levels (no problems, slight problems, moderate problems, severe problems, and unable to/extreme problems) and produces a total of 3125 (5^5^) health states (29, 30). The EQ VAS assesses the self-reported health status of subjects through a straight line (0: the worst health you can imagine; 100: the best health you can imagine). The reliability and validity of EQ-5D-5L have been verified in China (31). The EQ-5D-5L utility scores in this study were calculated using the China value set (32).

### HAQ-DI

The HAQ has two versions, the full HAQ and the short HAQ. The short HAQ which is used frequently contains the HAQ-DI, the VAS Pain Scale, and the VAS Patient Global. Further, the HAQ-DI which was developed by Stanford University in 1978 is often used by itself, particularly but not exclusively in the rheumatic disease (33). The HAQ-DI assesses a patient's level of functional ability with 20 questions in 8 categories, including dressing, rising, eating, walking, hygiene, reach, grip, and usual activities. Each category consists of 2 or 3 items and each item contains 4 level (0 means no difficulty, 1 means some difficulty, 2 means much disability, and 3 means unable to do). The score for each category is the highest score for the item in this category and the overall HAQ-DI score is the average of 8 categories, within 0 to 3 (33, 34).

### Data analysis

#### Descriptive statistics

Descriptive statistics (mean and standard deviation (SD) for continuous variables, frequency and percentage for categorical variables) were used for the sample characteristics. The distributions of EQ-5D-5L utility score and HAQ-DI score were showed through the figures.

#### Correlation test

The estimation of the mapping algorithm requires conceptual overlap between the source scale and the target scale (35–38). Spearman rank correlations were used to test the correlations between the HAQ-DI scores and EQ-5D-5L utility scores, so that we could ensure the degree of conceptual overlap. In addition, the correlations of different variables were tested by Spearman rank correlations to guarantee low collinearity among the independent variables included in the mapping algorithm. The strength of correlation could be divided to 4 level (very weak = 0–0.19; weak = 0.20–0.39; moderate = 0.40–0.59; strong = 0.60–0.79; and very strong = 0.80–1.00) (39, 40).

### Mapping model

Mapping consists of two broad approaches, direct mapping and response mapping. We used seven statistical models for developing a simpler and more accurate mapping algorithm, based on guidelines and previous researches (35). Ordinary least squares regression (OLS), the general linear regression model (GLM), MM-estimator model (MM), Tobit regression model (Tobit), Beta regression model (Beta) and the adjusted limited dependent variable mixture model (ALDVMM) were used for direct mapping and Multivariate Ordered Probit regression model (MV-Probit) for response mapping.

OLS, which assumes a linear relationship between the dependent variables and independent variables, is the most commonly used in direct mapping due to its simplicity (40, 41). But OLS performs poorly for predicting poor or full health, and the predicted values may be outside of the reasonable range (18, 36, 42). GLM, a flexible form of OLS, allows the outcome variables to have non-normal error distributions (43, 44). As one of robust regression technique, MM builds on minimizing some function of the residuals and a measure of dispersion of the residuals to achieve a high breakdown point with high efficiency (45–47). Given the EQ-5D-5L score has a ceiling effect (36, 43), and our measurements with a left-skewed distribution are clustered at 1, Tobit was used for mapping. Tobit can estimate the linear relationships among variables, as a censored model. But it is sensitive to violations of heteroscedasticity or non-normality (48, 49). Beta can avoid the predicted value fall outside of the reasonable range by setting the value of the dependent variable between 0-1. In addition, Beta is also suitable for heteroscedasticity or non-normality (50, 51). The following formula was used to adjust EQ-5D-5L scores to range of 0 to 1: *adjusted score* = *(original score*+*0.391)/1.391* (The range of EQ-5D-5L score is −0.391 to 1, based on the Chinese value set. If the original score was −0.391 or 1, the adjusted score was added or subtracted e^−12^ to ensure it fell between 0 and 1.). ALDVMM was developed as a mixture model for dealing with the distributional features of EQ-5D-3L. It could effectively capture the multimodal properties of EQ-5D score, boundary value and the gap between the value for full health and other health states (18). ALDVMM has been used in numerous previous researches (17, 52, 53), and confirmed is applicable to EQ-5D-5L (36, 52, 54–56). In response mapping, the dimension results obtained by the mapping algorithm are used to calculate the health utility values, based on the value set (36). Order Probit and Order Logit, etc., are commonly used models, but they are unable to explain the correlations between different dimensions. Multivariate ordered Probit method(MV-Probit) developed by Conigliani can solve the problem well (57).

### Variables

We chose the EQ-5D-5L total score and values of each dimension of EQ-5D-5L as the dependent variable for direct and response mapping, respectively. The HAQ-DI total score was included as the main independent variable for all mappings. The reason for we didn't chose the scores of different parts of HAQ-DI as independent variables for response mapping was that the sample size was slightly lacking. To obtain a more accurate mapping algorithm, we also included factors such as age, gender, BMI, DAS28-ESR, and PtAAP as independent variables based on the correlation between the EQ-5D-5L utility scores and each variable, as well as between the variables. It should be noted that we had scaled PtAAP (divided by 100) for calculation and presentation in the regression. And different combinations of variables were set in order to balance the accuracy, simplicity and generalizability of the mapping algorithms (Table 1).

**Table 1:** Combinations of variables.

**Combination**	**Explanatory variables**
Combination 1	HAQ-DI
Combination 2	HAQ-DI, PtAAP
Combination 3	HAQ-DI, Age, Gender
Combination 4	HAQ-DI, Age, BMI, Gender
Combination 5	HAQ-DI, DAS28-ESR, PtAAP, Age, BMI, Gender

HAQ-DI, the health assessment questionnaire disability index; PtAAP, the patient's assessment of arthritis pain; BMI, body mass index; DAS28, 28 joint counts; ESR, erythrocyte sedimentation rate.

### Validation and comparison of mapping algorithms

Given our sample was not rich, the bootstrap was used for validation of mapping algorithms (13). Firstly, a bootstrap sample of the same size as the original sample was drawn from the original sample. Secondly, mapping algorithms were fitted from the bootstrap sample using all statistics models, for all combinations of variables. Thirdly, the health utility values predicted using the mapping algorithms were compared with the observed health utility values, in the original sample. Finally, the mean of the health utility score predicted (MEAN-P), absolute difference between mean predicted and mean observed scores (ADM), mean absolute error (MAE), root mean square error (RMSE), adjusted *R*^2^ (adj*R*^2^) and concordance correlation coefficient (CCC) were calculated, recorded (43). After repeating the aforementioned four steps 500 times, the ranking of four indictors (MAE, RMSE, adj*R*^2^, CCC) were recorded and averaged for each combination. The mapping algorithm with the best average ranking was the optimal mapping algorithm in different combination of variables.

All statistical analyses were performed by stata15, programs R and Microsoft^®^ Excel 2019.

## Results

### Sociodemographic characteristics and patient-reported outcomes

**Figure 1:**
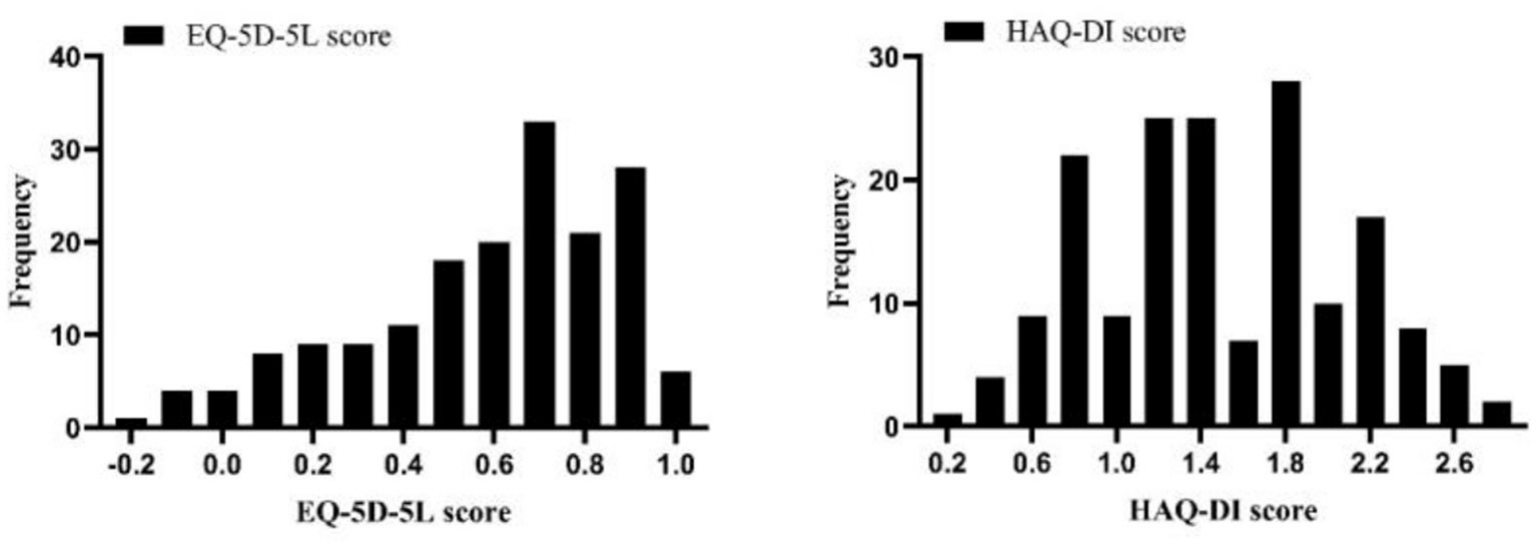
Distributions of EQ-5D-5L utility scores and HAQ-DI scores.

A total of 172 eligible patients were enrolled in the researches (Table 2). Their mean age (SD) was 50.82 (12.09) years, which was not significantly different from that of the general Chinese RA patient population (*p* > 0.1) (2, 26, 58). The proportion of female was 63.37%. A total of 126 health states were obtained and the mean scores (SD) were 0.59 (0.28) and 1.49 (0.60) for EQ-5D-5L utility score and HAQ-DI score, respectively. The distributions were left skewed and normally for EQ-5D-5L utility scores and HAQ-DI scores, respectively (Figure 1).

**Table 2:** Socio-demographic characteristics and patient-reported outcomes.

**Characteristics (*N* = 172)**	**Mean ±SD/N (%)**	**Median**	**Min**	**Max**
Age (years)	50.82 ± 12.09	53	20	70
**Gender**				
Male	63.00 (36.63%)			
Female	109.00 (63.37%)			
**Region**				
Urban	78.00 (45.35%)			
Rural	94.00 (54.65%)			
**Education**				
Below primary school	40.00 (23.26%)			
Primary school	35.00 (20.35%)			
junior middle school	27.00 (15.70%)			
High school/technical secondary school	40.00 (23.26%)			
Undergraduate/Junior college	21.00 (12.21%)			
Master degree or above	9.00 (5.23%)			
BMI	22.75 ± 3.72	22.30	14.64	35.67
EQ-5D-5L	0.59 ± 0.28	0.66	−0.19	1.00
EQ VAS	47.59 ± 19.85	48.20	10.00	90.00
PtAAP	63.85 ± 18.06	65.00	7.00	100.00
HAQ-DI	1.49 ± 0.60	1.50	0.25	2.88
DAS28-CRP	5.45 ± 1.20	5.45	2.01	8.32
DAS28-ESR	5.56 ± 1.37	5.66	1.77	9.09
SJC	14.04 ± 9.04	12.00	0.00	54.00
TJC	22.76 ± 14.44	19.00	0.00	68.00
ESR	48.63 ± 29.22	40.00	2.00	121.00
CRP	30.53 ± 36.31	13.00	0.50	177.10

SD, standard deviation; BMI, body mass index; EQ VAS, EuroQol visual analog scale; PtAAP, the patient's assessment of arthritis pain; HAQ-DI, the health assessment questionnaire disability index; DAS28, 28 joint counts; SJC, swollen joints count; TJC, tender joints count; ESR, erythrocyte sedimentation rate; CRP, high-sensitivity C-reactive protein.

### Correlation test results

Correlations between EQ-5D-5L utility score, HAQ-DI score, age, gender, BMI and DAS28-ESR, etc. were provided in Appendix 1 (see Supplementary material). There was a strong negative correlation between EQ-5D-5L utility score and HAQ-DI score (−0.7067). But other variables such as age, BMI and DAS28-ESR, etc. showed weak or moderate correlation with EQ-5D-5L utility score.

### Mapping algorithm performance

The results of mapping algorithms performance were presented in Table 3. A total of 35 mapping algorithms were fitted based on 5 combinations of variables and 7 statistics models. Limited the sample size and the convergence of the model, only the ALDVMM with one component was estimated.

**Table 3:** The results of mapping algorithms performance.

**Combination**	**Model**	**MEAN-P (SD)**	**ADM**	**MAE (SD)**	**GR (MAE)**	**RMSE (SD)**	**GR (RMSE)**	**adj*R*^2^ (SD)**	**GR (adjR^2^)**	**CCC (SD)**	**GR (CCC)**	**AGR**
Combination 1	OLS	0.5902 (0.0151)	0.0004	0.1552 (0.0025)	5	0.1994 (0.0011)	4	0.4907 (0.0060)	4	0.6640 (0.0169)	4	3
	GLM	0.5947 (0.0153)	0.0041	0.1668 (0.0026)	7	0.2092 (0.0011)	7	0.4396 (0.0070)	7	0.6017 (0.0181)	7	7
	MM	0.6060 (0.0169)	0.0155	0.1535 (0.0018)	3	0.2002 (0.0020)	6	0.4869 (0.0109)	6	0.6631 (0.0186)	5	6
	ALDVMM	0.5838 (0.0154)	0.0068	0.1553 (0.0033)	6	0.1981 (0.0015)	3	0.4972 (0.0078)	3	0.6579 (0.0191)	6	5
	Tobit	0.5925 (0.0152)	0.0019	0.1545 (0.0025)	4	0.1995 (0.0011)	5	0.4904 (0.0061)	5	0.6684 (0.0162)	3	3
	**Beta**	**0.5936 (0.0148)**	**0.0031**	**0.1497 (0.0024)**	**1**	**0.1951 (0.0014)**	**1**	**0.5128 (0.0071)**	**1**	**0.6937 (0.0153)**	**1**	**1**
	MV-Probit	0.5892 (0.0149)	0.0013	0.1511 (0.0030)	2	0.1954 (0.0012)	2	0.5110 (0.0064)	2	0.6766 (0.0172)	2	2
Combination 2	OLS	0.5904 (0.0150)	0.0002	0.1512 (0.0020)	6	0.1949 (0.0015)	4	0.5108 (0.0078)	4	0.6847 (0.0174)	5	5
	GLM	0.5953 (0.0149)	0.0047	0.1640 (0.0022)	7	0.2065 (0.0012)	7	0.4509 (0.0100)	7	0.6152 (0.0177)	7	7
	MM	0.6021 (0.0169)	0.0115	0.1505 (0.0018)	4	0.1957 (0.0025)	6	0.5065 (0.0130)	6	0.6878 (0.0196)	4	6
	ALDVMM	0.5844 (0.0146)	0.0062	0.1500 (0.0028)	3	0.1928 (0.0017)	3	0.5213 (0.0089)	3	0.6839 (0.0188)	6	3
	Tobit	0.5925 (0.0151)	0.0019	0.1505 (0.0020)	5	0.1949 (0.0015)	5	0.5107 (0.0078)	5	0.6887 (0.0169)	3	4
	**Beta**	**0.5952 (0.0141)**	**0.0047**	**0.1454 (0.0018)**	**1**	**0.1889 (0.0020)**	**1**	**0.5403 (0.0099)**	**1**	**0.7179 (0.0158)**	**1**	**1**
	MV-Probit	0.5887 (0.0146)	0.0018	0.1474 (0.0024)	2	0.1900 (0.0019)	2	0.5349 (0.0097)	2	0.6987 (0.0185)	2	2
Combination 3	OLS	0.5900 (0.0139)	0.0006	0.1455 (0.0030)	6	0.1829 (0.0013)	5	0.5666 (0.0064)	5	0.7328 (0.0121)	4	5
	GLM	0.5957 (0.0144)	0.0052	0.1586 (0.0029)	7	0.1966 (0.0015)	7	0.4993 (0.0131)	7	0.6637 (0.0151)	7	7
	MM	0.6125 (0.0169)	0.0219	0.1424 (0.0023)	3	0.1846 (0.0027)	6	0.5582 (0.0135)	6	0.7291 (0.0157)	6	6
	ALDVMM	0.5842 (0.0138)	0.0063	0.1450 (0.0033)	5	0.1811 (0.0017)	3	0.5749 (0.0083)	3	0.7309 (0.0131)	5	4
	Tobit	0.5912 (0.0140)	0.0007	0.1444 (0.0031)	4	0.1823 (0.0014)	4	0.5692 (0.0068)	4	0.7369 (0.0123)	3	3
	**Beta**	**0.5935 (0.0133)**	**0.0029**	**0.1390 (0.0033)**	**1**	**0.1777 (0.0016)**	**2**	**0.5909 (0.0078)**	**2**	**0.7617 (0.0101)**	**1**	**1**
	MV-Probit	0.5903 (0.0134)	0.0003	0.1406 (0.0035)	2	0.1777 (0.0015)	1	0.5909 (0.0070)	1	0.7506 (0.0114)	2	1
Combination 4	OLS	0.5897 (0.0134)	0.0009	0.1435 (0.0025)	5	0.1782 (0.0016)	5	0.5859 (0.0075)	5	0.7502 (0.0113)	4	5
	GLM	0.5955 (0.0139)	0.0050	0.1562 (0.0025)	7	0.1921 (0.0016)	7	0.5190 (0.0160)	7	0.6849 (0.0131)	7	7
	MM	0.6102 (0.0173)	0.0196	0.1413 (0.0022)	3	0.1803 (0.0036)	6	0.5763 (0.0180)	6	0.7451 (0.0167)	5	6
	ALDVMM	0.5836 (0.0143)	0.0069	0.1436 (0.0036)	6	0.1775 (0.0030)	3	0.5894 (0.0143)	3	0.7449 (0.0165)	6	4
	Tobit	0.5903 (0.0138)	0.0003	0.1424 (0.0027)	4	0.1777 (0.0018)	4	0.5882 (0.0088)	4	0.7544 (0.0120)	3	3
	**Beta**	**0.5936 (0.0129)**	**0.0031**	**0.1372 (0.0030)**	**1**	**0.1739 (0.0021)**	**2**	**0.6058 (0.0100)**	**2**	**0.7748 (0.0092)**	**1**	**1**
	MV-Probit	0.5902 (0.0129)	0.0004	0.1387 (0.0030)	2	0.1736 (0.0017)	1	0.6071 (0.0077)	1	0.7655 (0.0104)	2	1
Combination 5	OLS	0.5898 (0.0131)	0.0008	0.1360 (0.0027)	5	0.1709 (0.0019)	4	0.6147 (0.0088)	4	0.7756 (0.0110)	4	4
	GLM	0.5963 (0.0136)	0.0057	0.1539 (0.0026)	7	0.1888 (0.0021)	7	0.5295 (0.0240)	7	0.6993 (0.0129)	7	7
	MM	0.6110 (0.0183)	0.0204	0.1342 (0.0029)	3	0.1737 (0.0049)	5	0.6015 (0.0234)	5	0.7735 (0.0156)	5	5
	ALDVMM	0.5809 (0.0160)	0.0097	0.1434 (0.0081)	6	0.1784 (0.0090)	6	0.5791 (0.0438)	6	0.7401 (0.0372)	6	6
	Tobit	0.5888 (0.0134)	0.0018	0.1353 (0.0031)	4	0.1703 (0.0020)	3	0.6173 (0.0093)	3	0.7817 (0.0127)	3	3
	**Beta**	**0.5956 (0.0123)**	**0.0051**	**0.1264 (0.0034)**	**1**	**0.1644 (0.0028)**	**1**	**0.6433 (0.0123)**	**1**	**0.8034 (0.0086)**	**1**	**1**
	MV-Probit	0.5893 (0.0124)	0.0012	0.1283 (0.0036)	2	0.1645 (0.0021)	2	0.6429 (0.0092)	2	0.7944 (0.0102)	2	2

OLS, Ordinary least squares regression; GLM, the general linear regression model; MM, MM-estimator model; Tobit, Tobit regression model; Beta, Beta regression model; ALDVMM, the adjusted limited dependent variable mixture model; MV-Probit, Multivariate Ordered Probit regression model; MEAN-P, mean of the health utility score predicted; ADM, absolute difference between mean predicted and mean observed scores; GR, group ranking; MAE, mean absolute error; RMSE, root mean square error; adj*R*^2^, adjusted *R*^2^; CCC, concordance correlation coefficient; AGR, average group ranking. The best performers have been bolded.

The MEAN-P ranged from 0.5808 (ALDVMM of combination 5) to 0.6124 (MM of combination 3), where the OLS of combination 2 had the closest predicted score (ADM = 0.0002) to the observed mean score. The mapping algorithms of combination 5 had the lower MAE and RMSE and higher adj*R*^2^ and CCC as the number of variables increasing. Of all mapping algorithms, the range was 0.1263 (Beta of combination 5) to 0.1668 (GLM of combination 1) for MAE, 0.1644 (Beta of combination 5) to 0.2092 (GLM of combination 1) for RMSE, 0.4394 (GLM of combination 1) to 0.6432 (Beta of combination 5) for adj*R*^2^, 0.6016 (GLM of combination 1) to 0.8034 (Beta of combination 5) for CCC.

### Best performing mapping algorithm

**Figure 2:**
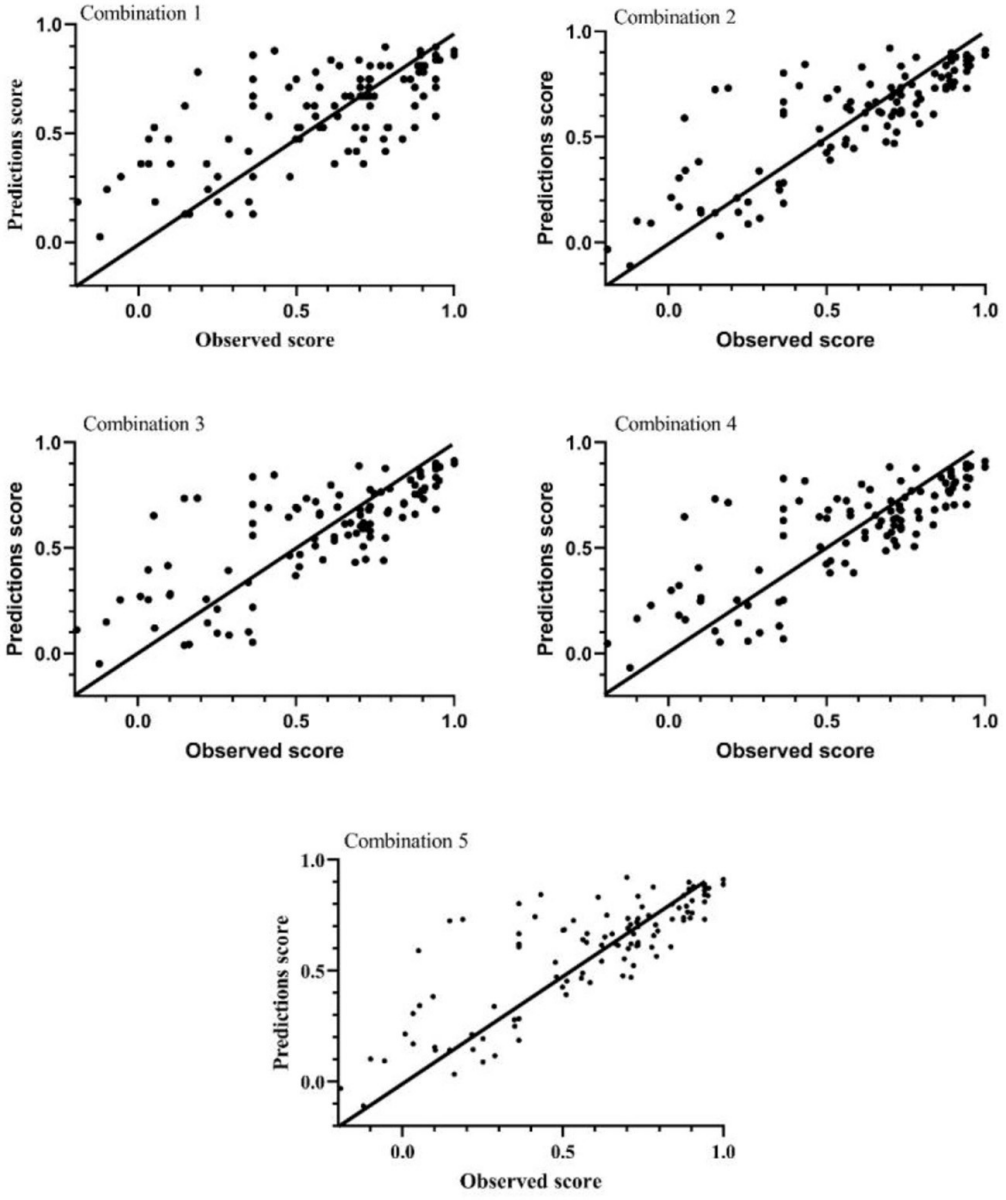
The observed and predicted EQ-5D-5L utility score.

The average group ranking (AGR) showed that Beta performs the best, followed by MVROD-Probit. It should be note that the AGR of MV-Probit and Beta both are 1 for combination 3 and 4. The Beta performed better in MAE and CCC, but worse in RMSE and adjR^2^ than MVROD-Probit. In addition, compared with Beta, MVROD-Probit performed better in ADM for each combination. But Beta performed the best in MAE, RMSE, adjR^2^ and CCC for combination 1, 2, and 5. Based on these results, we thought that the best performing mapping algorithm was the one constructed based on the Beta, for each combination. As for the best combination, the results showed that the more variables incorporated, the better the mapping algorithm performed. But we did not think the combination 5 was the best because it required more variables. The reality was that we may could not obtain the data for these variables. Thus, we recommended choosing the combination according to the actual available data and selecting the mapping algorithm constructing based on Beta. Figure 2 demonstrated the consistency between the observed EQ-5D-5L utility score and predicted EQ-5D-5L utility score, based on Beta, for each combination. The Pearson correlation coefficients were 0.6228, 0.6411, 0.6889, 0.6975, and 0.7314 for combination 1 to 5, respectively. They indicated a high correlation between the observed EQ-5D-5L score and predicted EQ-5D-5L score.

### The mapping algorithm parameters

Table 4 presents the parameters of mapping algorithms constructed based on Beta for predicting EQ-5D-5L utility scores from HAQ-DI scores. For all combinations, HAQ-DI score, gender, BMI and PtAAP were significant predictors of EQ-5D-5L utility scores, but age and DAS28-ESR were insignificant predictors. According to the formula (see Methods) for adjusting EQ-5D-5L scores, the mapping algorithms formula can be showed as following:


$$\begin{array}{l} EQ-5D-5L\;score=1.391\times \frac{e^{constant+\sum_{1}^{i}\beta_{i}X_{i}}}{1+e^{constant+\sum_{1}^{i}\beta_{i}X_{i}}}-0.391 \end{array}$$

**Table 4:** The parameters of mapping algorithm from EQ-5D-5L to HAQ-DI based on beta.

	**Combination 1**	**Combination 2**	**Combination 3**	**Combination 4**	**Combination 5**
Constant	3.02428^***^	3.48797^***^	3.68661^***^	4.35968^***^	**4.93095** ^***^
HAQ-DI	−1.34965^***^	−1.26531^***^	−1.32507^***^	−1.32570^***^	**-1.22172** ^***^
Gender			−0.68938^***^	−0.71406^***^	–0.76041^***^
Age			−0.00473	−0.00300	–**0.00137**
BMI				−0.03246^*^	–0.02798^*^
PtAAP		−0.90418^**^			–1.11943^***^
DAS28-ESR					–**0.02660**

^*^p < 0.1, ^**^p < 0.05, ^***^p < 0.01.

The PtAAP scores used here should be the scaled scores (divided by 100).

HAQ-DI, the health assessment questionnaire disability index; PtAAP, the patient's assessment of arthritis pain; BMI, body mass index; DAS28, 28 joint counts; ESR, erythrocyte sedimentation rate. The best performers have been bolded.

[*i* is the count of independent variables included, β_*i*_ is the coefficient (parameters showed in Table 4), *X*_*i*_ is the independent variable, such as HAQ-DI, gender, age, etc.].

## Discussion

This research developed the mapping algorithms for estimating EQ-5D-5L utility scores from HAQ-DI scores in Chinese RA patients. Up to now, two researches have constructed the mapping algorithm from HAQ-DI to EQ-5D-5L based on Chinese RA patients, of which Thoma's research (13) used the Beta and MV-Probit and Dexin ZHOU's research (12) used OLS and Tobit. But they used different statistical models and, respectively concluded that OLS (Dexin ZHOU) and MVROD-Probit (Thomas) were the best models for developing the mapping algorithms. This was difficult for researchers to choose the optimal mapping algorithm to conduct relative researches. Furthermore, ALDVMM was confirmed by several studies to have significant advantages in predicting EQ-5D-5L utility scores (36, 55, 59). Ducournau P indicated that the relationship between HAQ-DI scores and EQ-5D utility scores was non-linear in his study (21). Based on these results, this research incorporated additional models to construct mapping algorithms, including OLS, GLM, MM, ALDVMM, Tobit, Beta, and MVROD-Probit. Compared with the single sample source of previous two researches, this research increased to four sample sources, although also not particularly rich in sample size. In addition, the variables included in this research were richer than previous researches which only includes HAQ-DI score, Pain VAS (equivalent to PtAAP), EQ-VAS and PrGA (equivalent to PhGADA). The mapping algorithms in this research were constructed after considering linear model, mixed model, response mapping and a large number of variables. It was benefit for obtaining the health utility values of Chinese RA patients, and then conducting pharmacoeconomic evaluation to enhance the efficiency of healthcare resource allocation.

The selection of variables referred to the existing literature and the correlations test results (Appendix 1). Among the previous researches that develop the mapping algorithms from HAQ-DI to EQ-5D-5L, researchers often included HAQ-DI score, HAQ-DI item score, Pain-VAS or DAS28 as explanatory variables (14, 15, 20, 60). Some researches would also include some demographic indicators as explanatory variables, like age and gender. However, we found that not only age and gender will affect the health utility values of RA patients, but also BMI will affect them. To enhance the accuracy of the mapping algorithm, we also tried to incorporate some clinical indicators, such as ESR, CRP, DAS28, PhGADA, PtAAP, and PtGADA. The correlations test showed that EQ-5D-5L utility score had a strong negative correlation with the HAQ-DI score (−0.7067), a moderate negative correlation with PtAAP (−0.4040) and PtGADA (−0.4166), and a weak correlation with age, BMI, ESR, CRP, DAS28-ESR, DAS28-CRP, TJC, SJC, etc. Given the correlation between PtAAP and PtGADA was strong, and the correlation between PtGADA and EQ-5D-5L score was stronger than that between PtAAP and EQ-5D-5L score, we included the PtGADA as an explanatory variable. By the same token, we chose DAS28-ESR rather than DAS28-CRP. Due to the DAS28 was calculated by ESR, CRP, TJC, and SJC, we excluded the four indicators. Finally, HAQ-DI score, gender, age, BMI, PtGADA and DAS29-ESR were included as explanatory variables. Although the results showed that the more variables included, the more accurate the mapping algorithm was, we still recommended that researchers to choose a mapping algorithm based on actual data. And we provided mapping algorithms for different combinations of variables.

Among the seven statistics models we used, Beta model performed the best, followed by the MVROD-Probit. Some researches had presented that direct mapping with mixture model was better than direct mapping with linear regression, better than the response mapping (36, 52). ALDVMM was such a mixture model which could effectively capture the multimodal properties of EQ-5D utility score (18). In this research, however, ALDVMM had not performed as well as expected. The ALDVMM with 2 or more components suffered from the problem of non-convergence, which may change as the simple size increases. About MVROD-Probit, the results of Thomas' research showed that MVROD-Probit performed better than Beta, which was different from our results (13). Although our sample size was larger than that of Thomas' research, given the gap between the two sample size was small and our sample did not cover all the health status of patients, we thought a larger sample with more health status was necessary to demonstrate the difference of the two models.

Several limitations may affect the representativeness of our results. Firstly, despite the relatively rich source of the sample, the small sample size affected the using of model, like ALDVMM and MVROD-Probit, and increased the uncertainty of the results. Secondly, although the bootstrap was used to provide an assessment of the internal validity of the mapping procedures considered in this research, we could not verify the external validity of the mapping algorithm. Thirdly, most early RA patients have no typical clinical symptoms (61), and the treatment and economic evaluation mainly occur in these patients (62, 63), in China. Thus, our sample only included RA patients with middle and advanced stage, which may cause bias in predicting health utility values for patients with early stage. Finally, no patients chose 5 at mobility and anxiety/depressions dimensions of EQ-5D-5L in our sample, which may lead to bias for the results of response mapping.

## Conclusion

More models and variables were used to construct the mapping algorithms in this research. And we found that mapping algorithms based on Beta performed better. Also, the more variables included, the mapping algorithm performed more better. Researchers could reasonably choose the combinations of variables and the mapping algorithm recommended based on the actual available data. These mapping algorithms could help researchers to obtain the health utility values of Chines RA patients and thus conduct other studies, like pharmacoeconomic evaluation.

## Data availability statement

The raw data supporting the conclusions of this article will be made available by the authors, without undue reservation.

## Ethics statement

The studies involving human participants were reviewed and approved by Clinical Trial Ethics Committee of Huashan Hospital Affiliated to Fudan University (Reference Number 2019-252). The patients/participants provided their written informed consent to participate in this study.

## Author contributions

CW, QW, and XX made their contributions to the conception and design of the work. CW, YH, and QW made their contributions to the acquisition and analysis of the data. ZX and YH made their contributions to the interpretation of data. CW made contributions to drafting of the work. CW, ZX, and XX made their contributions to revision of the work. All authors of this work has approved the submitted version and have agreed both to be personally accountable for the author's own contributions and to ensure that questions related to the accuracy or integrity of any part of the work.
