# Supplementary material for: Mapping health assessment questionnaire disability index onto EQ-5D-5L in China
Source: Front Public Health. 2023 Apr 18;11:1123552. doi: 10.3389/fpubh.2023.1123552 (PMC10151687; doi:10.3389/fpubh.2023.1123552)
Supplement: Supplementary file 1 [file Table_1.DOCX]

## Supplementary Material

**Appendix 1 The Spearman's correlation coefficient between variables**

| Variables | EQ-5D-5L | Age | Gender | BMI | Region | Education | HAQ-DI | SJC | TJC | ESR | CRP | DAS28-CRP | DAS28-ESR | PtAAP | PhGADA | PtGADA |
| --- | --- | --- | --- | --- | --- | --- | --- | --- | --- | --- | --- | --- | --- | --- | --- | --- |
| EQ-5D-5L | 1 |  |  |  |  |  |  |  |  |  |  |  |  |  |  |  |
| Age | -0.2046** | 1 |  |  |  |  |  |  |  |  |  |  |  |  |  |  |
| Gender | -0.3176** | -0.1340 | 1 |  |  |  |  |  |  |  |  |  |  |  |  |  |
| BMI | -0.1752* | 0.0764 | -0.1058 | 1 |  |  |  |  |  |  |  |  |  |  |  |  |
| Region | -0.1864* | 0.4007** | -0.0203 | -0.0062 | 1 |  |  |  |  |  |  |  |  |  |  |  |
| Education | 0.2743** | -0.5726** | -0.1489 | -0.0154 | -0.7128** | 1 |  |  |  |  |  |  |  |  |  |  |
| HAQ-DI | -0.6998** | 0.2232** | 0.0518 | 0.0387 | 0.1158 | -0.1889* | 1 |  |  |  |  |  |  |  |  |  |
| SJC | -0.0968 | 0.1140 | 0.0571 | -0.112 | -0.0536 | -0.0224 | 0.2169** | 1 |  |  |  |  |  |  |  |  |
| TJC | -0.1983** | 0.1887* | 0.1091 | -0.0113 | 0.0398 | -0.1347 | 0.3226** | 0.6826** | 1 |  |  |  |  |  |  |  |
| ESR | -0.2148** | 0.1701* | 0.2829** | -0.0393 | 0.0792 | -0.1438 | 0.1499* | 0.3251** | 0.2904** | 1 |  |  |  |  |  |  |
| CRP | -0.0024 | -0.0672 | -0.1365 | -0.0145 | -0.0670 | 0.1317 | 0.1982** | 0.1971** | 0.1260 | 0.5179** | 1 |  |  |  |  |  |
| DAS28-CRP | -0.1533* | 0.0751 | 0.0115 | -0.0553 | 0.0132 | -0.0371 | 0.2337** | 0.6687** | 0.6748** | 0.3702** | 0.4330** | 1 |  |  |  |  |
| DAS28-ESR | -0.2332** | 0.0815 | 0.2223** | -0.0650 | 0.0813 | -0.1518* | 0.1369 | 0.6306** | 0.6479** | 0.5024** | 0.1589* | 0.8384** | 1 |  |  |  |
| PtAAP | -0.3731** | 0.1746* | -0.0856 | 0.1080 | 0.0157 | -0.0479 | 0.3860** | 0.1512* | 0.1878* | 0.2331** | 0.2817** | 0.3167** | 0.1213 | 1 |  |  |
| PhGADA | -0.2040** | 0.1887* | -0.0539 | 0.0501 | 0.0294 | -0.1034 | 0.3267** | 0.2948** | 0.3066** | 0.2809** | 0.3062** | 0.3222** | 0.2033** | 0.6067** | 1 |  |
| PtGADA | -0.4018** | 0.1772* | -0.0692 | 0.1331 | 0.0653 | -0.0814 | 0.4313** | 0.1826* | 0.1992** | 0.3089** | 0.2774** | 0.3429** | 0.1801* | 0.8169** | 0.5678** | 1 |

Note: **p*<0.05, ** *p*<0.01; SJC: swollen joints count; TJC: tender joints count; ESR: erythrocyte sedimentation rate; CRP: high-sensitivity C-reactive protein; DAS28: 28 joint counts; PtAAP: the patient's assessment of arthritis pain; PtGADA: the patient's global assessment of disease activity; PhGADA: the Physician’s global assessment of disease activity.
